# Blackleg in Cattle in the Irkutsk Region

**DOI:** 10.3389/fvets.2022.872386

**Published:** 2022-05-12

**Authors:** Andrei A. Blokhin, Nadezhda N. Toropova, Olga A. Burova, Ivan V. Iashin, Olga I. Zakharova

**Affiliations:** Federal Research Center for Virology and Microbiology, Branch in Nizhny Novgorod, Nizhny Novgorod, Russia

**Keywords:** cattle, blackleg, outbreak, pathological, anatomical changes, seasonality

## Abstract

Blackleg is an acute, toxic, infectious, non-contagious disease of domestic and wild ruminants that occurs while the animals are pastured. This article describes an outbreak of blackleg on a farm in Siberia (Russia) in 2019. We provide a detailed description of the cases based on the results of comprehensive diagnostic and epidemiological investigations. For description of case and evaluation, we used the following methods: owner observations, descriptive epidemiology, clinical diagnostics, pathological examination and bacteriology. The distinctive features (in addition to the characteristic features) were as follows: the outbreak of the disease occurred in early spring when there was abundant snow cover and under unfavorable living conditions of animals and traumas; the disease appeared in both vaccinated and unvaccinated cattle; the characteristic clinical signs were low-grade fever, the absence of crepitus, and the presence of haematomas containing erythrocytes with basophilic granularity; thrombs in vessel and vacuolization in tissue of the adrenal gland. This paper aimed to present classical and new clinical and pathology changes in cattle with blackleg in winter conditions of Russian Siberia.

## Introduction

Blackleg is an acute non-contagious, toxic, infectious disease of livestock and wild animals that is characterized by fever, myositis, systemic infection and rapid mortality. The causative agent of the disease is the obligate anaerobic bacillus *Clostridium chauvoei*, which persists in soil in the form of resistant spores (1–3). This creates convenient conditions for the formation of endemic zones (4, 5).

Contact of an animal with contaminated soil, water or food leads to the development of the disease (6). According to the common understanding of the pathogenesis of this disease, *C. chauvoei* spores in contaminated pastures undergo one or more cycles of replication in the intestinal tract without causing the clinical symptoms of the disease (7–9). When an animal experiences an injury that breaks the barrier of the skin and that open wound becomes contaminated with spores, the spores enter an anaerobic environment and germinate. The vegetative form of the pathogen releases toxins that cause the clinical manifestations of the disease, ultimately leading to the death of the host (10, 11).

Unvaccinated cattle aged 3 months to 4 years, buffaloes, sheep, goats, elk and reindeer are susceptible to blackleg (5, 12).

Blackleg is characterized by various disease courses, and the duration and frequency of outbreaks vary (13–16). The disease usually occurs in cattle in the summer and autumn. Poor fodder, including dry grass, increases the risk of the occurrence of an outbreak of blackleg (5).

Cases in animals are registered in dozens of countries around the world (Kazakhstan, USA (California), Austria, Algeria, Pakistan, Taiwan, Zambia, Belarus) (17–19).

In the Russian Federation, cases of blackleg have been registered each year in various regions (in the Irkutsk Region in 2011, Kursk Region in 2012, Chuvash Republic in 2013, Trans-Baikal Territory in 2014) (20) during grazing seasons, manifesting as sporadic cases or small outbreaks and only rarely as an enzootic disease.

In the acute form, the body temperature of the animal increases to 41–42°C; the animal shows signs of depression, including a refusal to eat and lethargy; and subcutaneous gas oedema forms, especially in the extremities, with swelling and crepitus in the affected muscles (21, 22). In the hyper-acute form, the clinical signs are usually not observed due to the sudden death of the affected animal (5).

The characteristic pathological and anatomical changes are acute necrohemorrhagic emphysematous myositis of the skeletal muscles, most often of the thoracic and pelvic belt muscles (5, 17, 18); expressed pulmonary oedema; myocarditis and petechial hemorrhages on the heart; liver enlargement; and venous hyperaemia of the liver and spleen (17, 22, 23). Visceral myonecrosis is less commonly noted and may affect the heart, hyoid muscles and diaphragm (18, 24). Fibrinous pericarditis occurs very rarely. There are descriptions of damage caused by *C. chauvoei* in the spleen, liver and kidneys. The spleen severely increased in size, congested and has dark black color. Microscopic studies shower leukocytes infiltrations in sinusoidal spaces and hemorrhages in the liver. Histological sections of kidneys shower hemorrhages, degeneration of renal tubules, necrosis of renal tubules, increased urinary spaces and necrosis of tubular epithelial cells (25). Emphysematous fetuses with lesions of the skeletal muscles and myocardium similar to those observed on the placenta are found in the uterus in cases of intrauterine infection (10).

The diagnosis has to be confirmed with laboratory tests. The bacteriological diagnosis of blackleg is based on the results of seeding on a nutrient growth medium, microscopy of smears, isolation of pure cultures and bioassays. Blackleg should primarily be differentiated from malignant oedema and anthrax (26–28).

The control of blackleg is based on symptomatic treatment and vaccination (29–33).

The aim of this study was to investigate the possible routes of pathogen entry into the animals, features of the pathogenesis of the disease, clinical symptoms and pathological changes in an outbreak of blackleg that occurred in March 2019 in Siberia. In this article, we analyzed the conditions and clinical and epidemic parameters of an outbreak of emphysematous carbuncle that occurred outside the usual season for this disease.

## Materials and Methods

Area: The field research was carried out from March to April 2019 in Zabitui village, Irkutsk region (E 102.818055; N 53.264713), and the laboratory research was performed in the Federal Research Center for Virology and Microbiology (Pokrov).

For data collection and evaluation, we used the following methods: owner observations, descriptive epidemiology, clinical diagnostics, pathological examination and bacteriology.

Epidemiological data included the size and type of herd, feeding and housing conditions of the cattle, and list of treatments. We assessed the conditions in which the livestock and the animals were kept during the outbreak based on the results of a survey conducted of the farm's facilities.

The retrospective analysis was based on available official data on blackleg outbreaks in cattle in the Russian Federation, Irkutsk region and Zabitui village over the past 10 years.

Symptoms and pathological changes were described in accordance with the standard guidelines for animal examinations and necropsy with the visual assessment of pathological changes. Necropsy of the carcasses was carried out in the area specifically allocated for the cremation of the remains. Clinical and pathological changes were studied in each adult diseased animal (*n* = 43). Smears of infiltrates sampled from swollen sites in 12 animals were stained with the Romanowsky-Giemsa stain and studied under microscopy at 650X magnification.

Samples for histological study were collected from 10 dead animals. Samples of heart, and of a variety of other tissues including liver and adrenal gland, had been collected in most cases and fixed in 10% buffered formalin (pH 7.2) for 24–72 h. All tissues had been processed by routine histologic techniques to produce 4-μm thick sections, which were stained with hematoxylin and eosin (H&E).

Laboratory diagnostics was carried out for all dead animals (*n* = 43) and three stillborn calves.

The laboratory diagnosis was based on:

- morphology: direct microscopic examination of cultures of *C. chauvoei* after Gram staining;
- microbiological purity: isolation on Kitt-Tarozzi medium and incubation in aerobic and anaerobic conditions;
- multiplex PCR-test.

The strains were identified based on cultural and morphological characteristics using classic tests (34). C. *chauvoei* were cultivated under standard anaerobic conditions at 37°C on Kitt-Tarozzi medium.

Samples positive for *C. chauvoei* were analyzed for *C. chauvoei* and *C. septicum* using multiplex PCR techniques (35).

Formal ethical review was not required for this study, according to local and national legislation, because this work did not involve any experiments on animals. Biological specimens for of were collected using standard procedures followed the guidelines of the State Veterinary Service of the Russian Federation. Farmers verbal consented for the participation of their animals in this study. In this study, animals were not killed for the purpose of research.

## Results

### Animal Welfare and Husbandry

The farm is located 800 meters southeast of Zabitui village, Alarsky district, Irkutsk region. As of March 16, 2019, the following animals were present: 108 cattle, 45 horses, and 250 sheep. Different species of animals were kept separate from each other on permanent flooring of straw, which was not removed, but from time to time was laid on top of snow and manure. The animals did not graze because there was a deep of snow on the pastures.

The animals were fed locally produced hay and crushed grains. Water for household needs and animal consumption was supplied from a borehole. The territory of the farm was established by a solid wooden fence and metal gates. The territory was used for logging and other economic activities. The territory was full of wooden rubbish and metal objects.

We conducted a study that excluded alimentary causes and poisoning with toxins. The study included toxicological analysis for mycotoxins, heavy metals, pesticides.

### Epidemiological Data

Official reports from the past 10 years yielded no records of blackleg in Zabitui village. This outbreak was the first to be registered in the area.

The outbreak started on March 17, 2019, with sudden death of three cattle on the second day after vaccination against blackleg. The animal carcasses were disposed of the same day by cremation in a specifically designated are.

The epidemic involved pregnant cows, cows with calves and young cattle (*n* = 43). They were vaccinated against blackleg and anthrax 2 days before the outbreak (15 March). Infections in other species, such as sheep, horses and buffaloes, that were vaccinated against blackleg in December 2018 were not observed.

**Figure 1:**
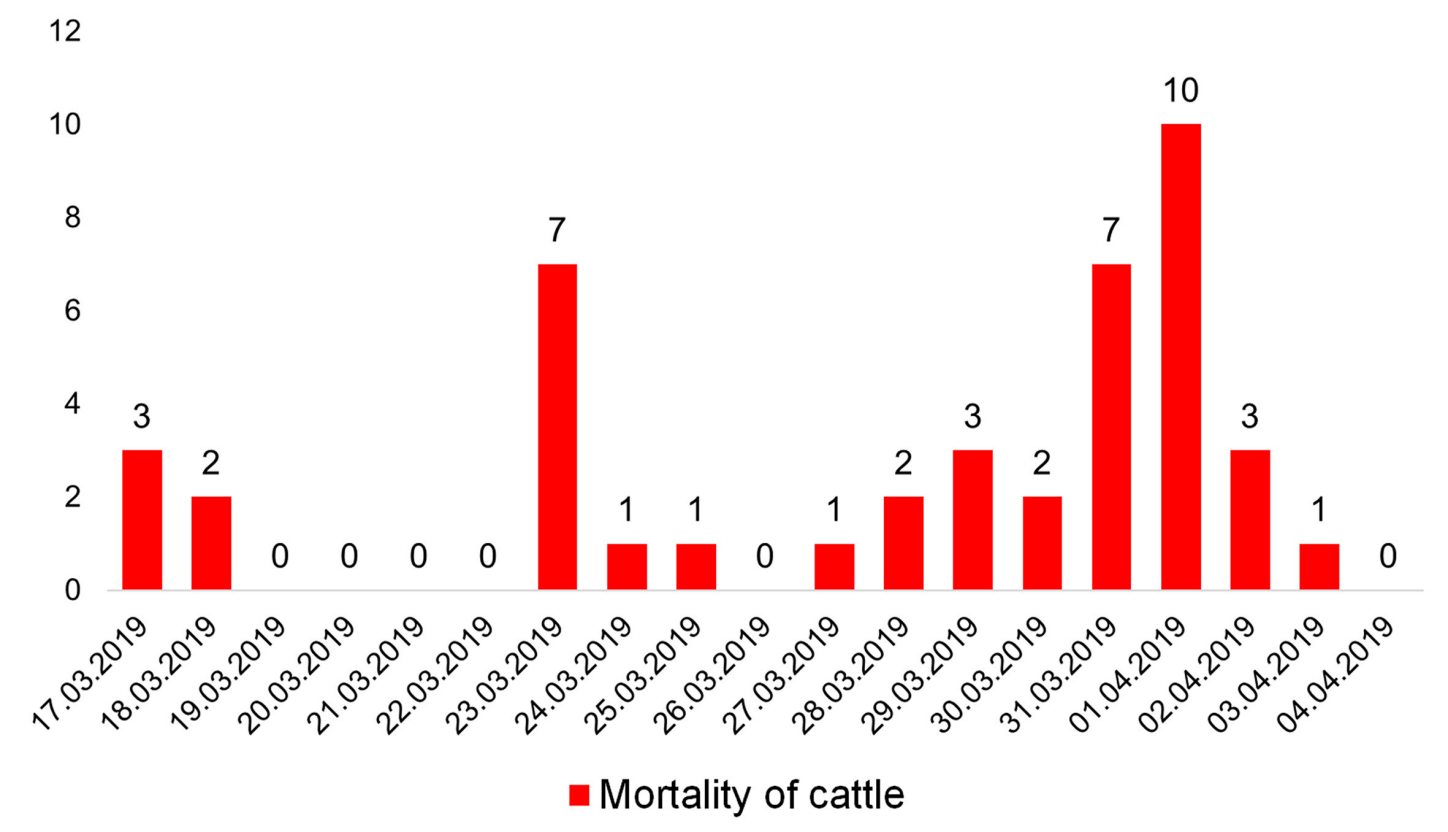
Animal deaths from 17 March to 04 April 2019.

The outbreak lasted 2 weeks, from March 17 to April 04, 2019. During this period, 43 head of cattle died (Figure 1). All of these animals were clinically and pathologically studies.

### Clinical Signs

**Figure 2:**
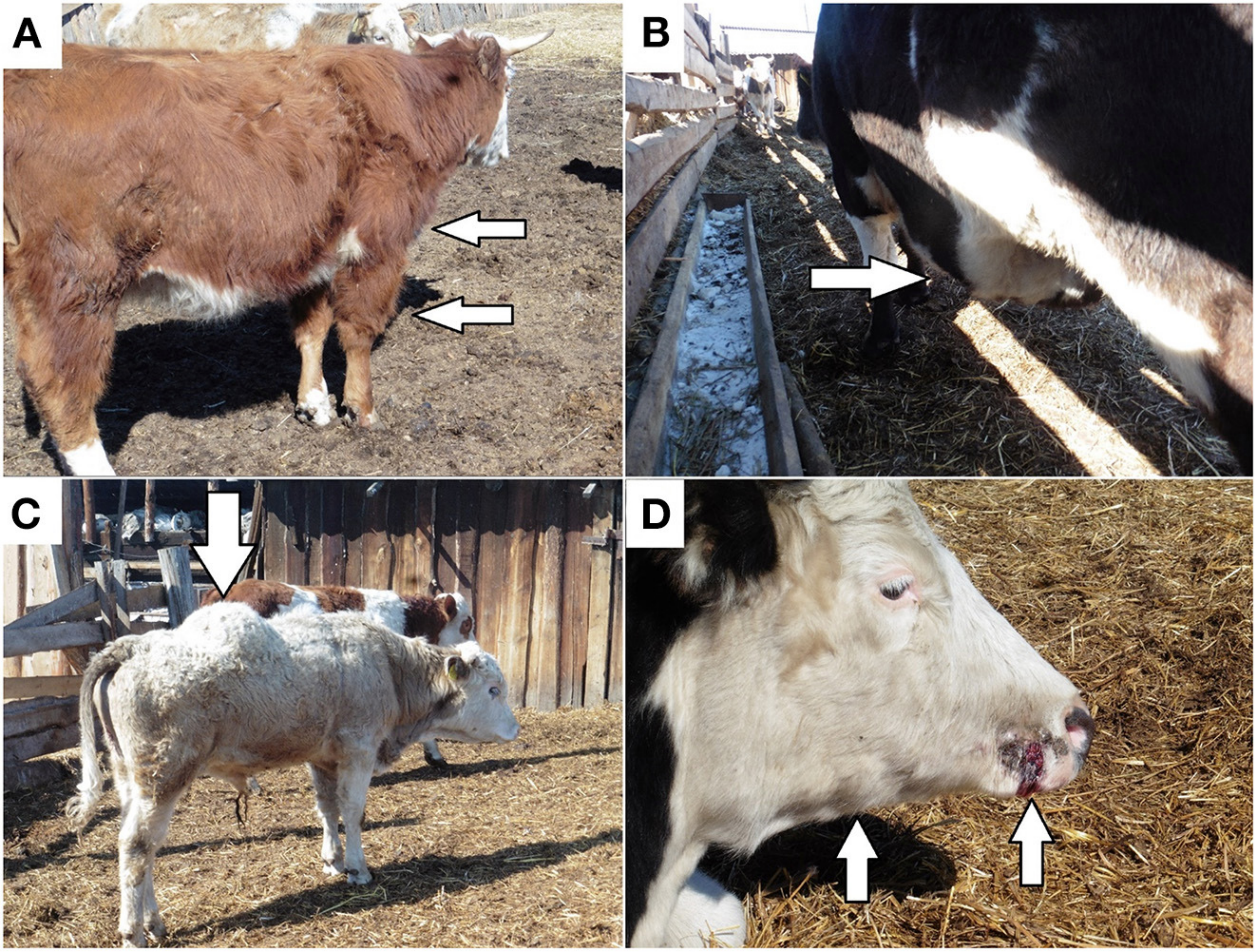
Distinct clinical signs of blackleg: **(A)** oedema in the scapular shoulder joint of the anterior right limb; **(B)** oedema in the abdominal cavity; **(C)** oedema in the back; **(D)** papilloma trauma on the cow muzzle with subsequent oedema and the formation of nodes in the subcutaneous tissue in the area of the right branch of the jaw.

The following clinical signs were observed in all affected animals: depression behavior, anorexia, and a high body temperature reaching 38.0–39.5°C. Gross swelling was present on the surfaces of the body, although no crepitus was palpable. In different animals, the oedema was localized to the limbs (Figure 2A), chest, abdomen (Figure 2B), and back (Figure 2C). Paracentesis of the oedema revealed fluid accumulation and hemorrhage. During abdominal auscultation, there were signs of increased gas formation in the intestine.

Several animals showed signs of papillomatosis on the muzzle and vulva, as well as birth trauma. The papilloma and birth trauma were accompanied by the subsequent formation of oedema and compaction of the surrounding subcutaneous tissue and muscles with the formation of dense nodes (Figure 2D).

Abortions and stillbirths were reported in pregnant cows. A total of 3 cows aborted, and all of them subsequently died.

### Autopsy

**Figure 3:**
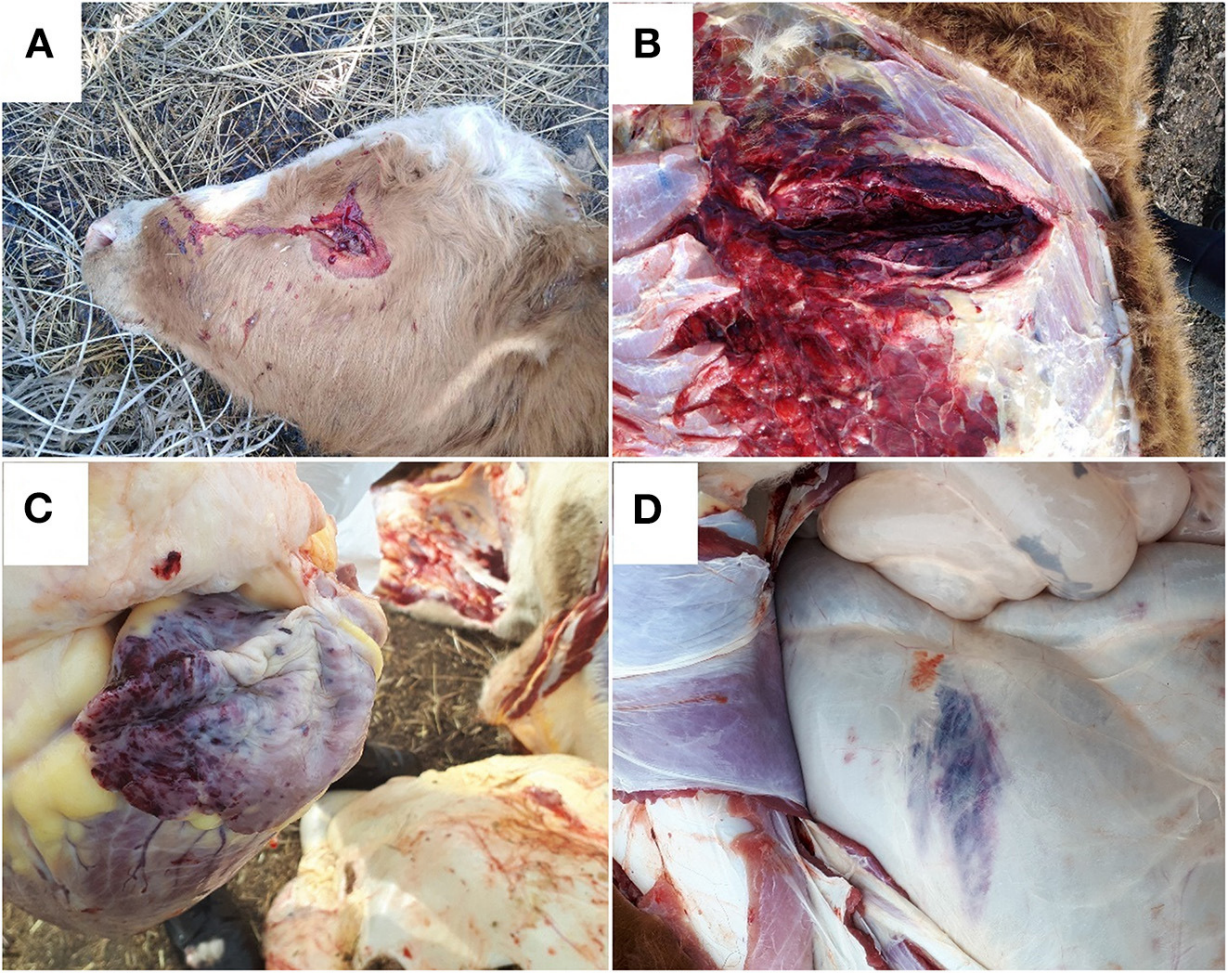
Pathological and anatomical changes in cattle with blackleg: **(A)** foamy, bloody liquid from the eyes; **(B)** haemorrhagic-necrotic myositis; **(C)** gross hemorrhage in the heart; **(D)** hemorrhage in the serous membrane of a scar.

Immediately after death, the bodies of the animals released foamy, bloody fluid from the mouth, nose and eyes (Figure 3A).

Necropsies were performed on all the carcasses of the cows and calves, and the following distinct changes were observed at the sites of oedema: haemorrhagic-necrotic myositis with gas formation and serous haemorrhagic infiltration of the loose subcutaneous tissue adjacent to muscles (Figure 3B).

Some animals had serous haemorrhagic lymphadenitis of the pulmonary lymph nodes (21; 48.8%), cardiac hemorrhage (11; 25.6%) (Figure 3C), ruminal tympany with abdominal anemia and thoracic hyperaemia (5; 11.6%), hemorrhage in the serous membrane of a scar (11; 25.6%) (Figure 3D), and hemorrhage in the subcutaneous tissue in the area of ribs 5–8 and 10–12 on the left side of the body (1; 2.3%).

In tweel animals in the region of the lumbar spine and on the abdominal side, there was a haematoma with coagulated blood that was approximately 25 cm long (along the spine) and approximately 13 cm wide. Yellow gelatinous infiltration was found in the muscular tissue of the right hind limb.

One animal had connective tissue adhesions between the peritoneum and the serous membrane of the intestine and between the pulmonary and bone pleura and hemorrhages on the abdominal wall. The is chronic changes, likely unrelated to the outbreak.

The necropsy of the stillborn calves revealed hemorrhages on the spleen, gross hemorrhages in the heart and a lack of any border between the cortical and medulla matter in the kidneys.

### Microscopic Pathology

**Figure 4:**
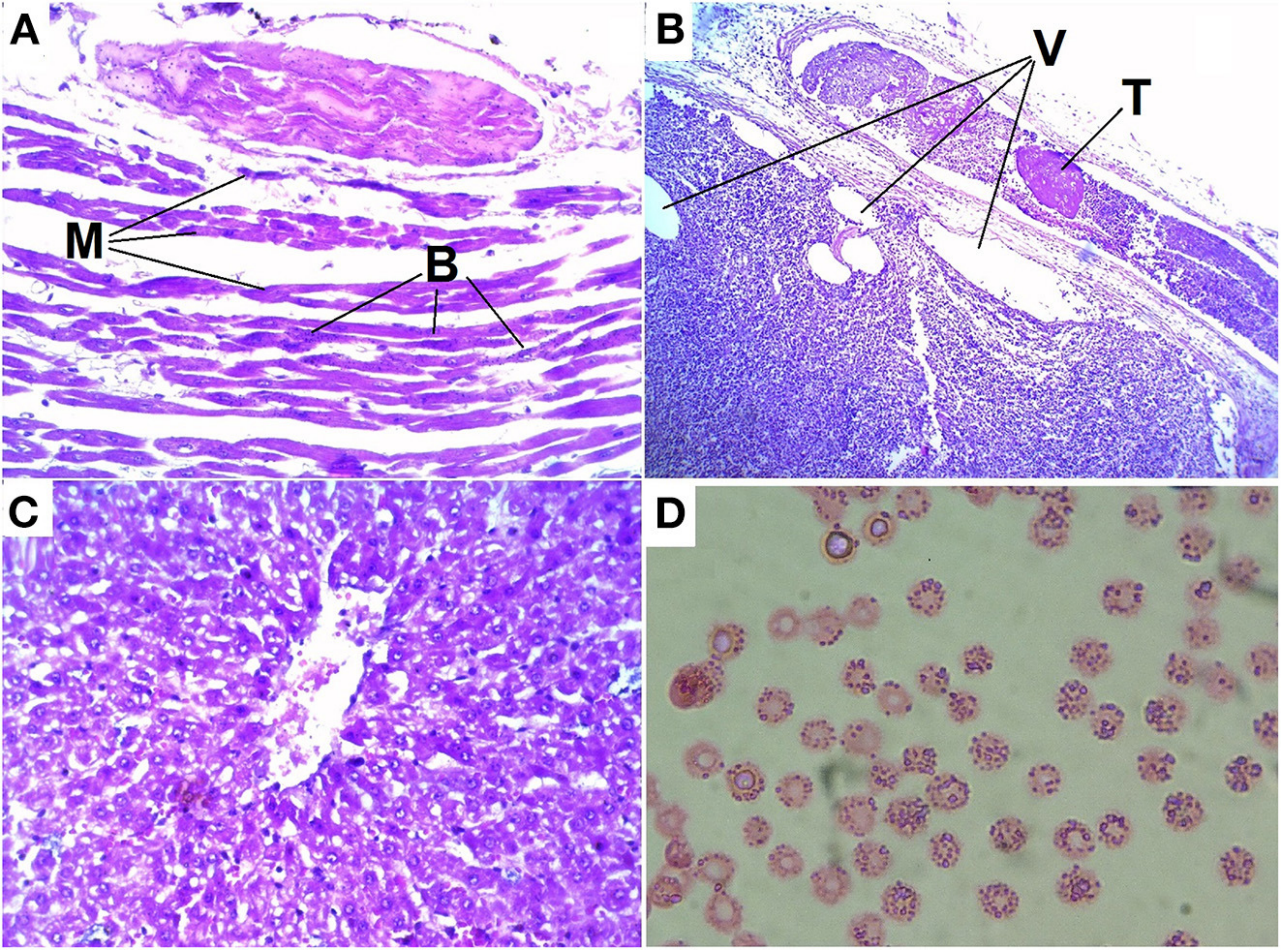
Histologic lesions of blackleg in cattle: **(A)** destruction, hypereosinophilia, loss of cross-striations, contraction bands and fragmentation of myocytes (M) of the heart and accumulations of bacteria (B); **(B)** thromb in vessel (T) and vacuolization (V) in tissue of the adrenal gland; **(C)** degenerative changes and vacuolization of hepatocytes; **(D)** basophilic granularity in cow red blood cells.

Myocytes of heart showed degenerative and/or necrotic changes consisting of eosinophilic, swollen, and fragmented muscle fibers that may have hypercontraction streaks and loss of striation. There was no leukocyte infiltration. In the interstitium, a multifocal accumulation of rods with rounded ends was found, some of them with a central or subterminal spore; these rods were observed singly or in clusters. Multifocal, there was interstitial hemorrhage in several areas; erythrocytes were observed between myofibrils. The interstitium was enlarged due to hemorrhages and proteinaceous edema (Figure 4A).

In the described case, characteristic vascular fibrinoid degeneration and thrombi were observed in the vessels of the heart and adrenal glands. In addition, degenerative changes and tissue vacuolization are found in the adrenal glands (Figure 4B).

In the liver, we observed degenerative changes and vacuolization in hepatocytes (Figure 4C).

In a microscopic examination of a blood smear obtained from a haematoma of the scapular shoulder joint that had been present for a week, erythrocytes with basophilic granularity were detected (Figure 4D). Inclusion basophilic granularity were observed in almost 100% of the cells.

### Laboratory Research

During testing of 46 samples (43 from animals and 3 stillborn calves), a large amount of gas on the Kitt-Tarozzi medium was observed only on the first day.

In the preparations of bacterial cultures on the Kitt-Tarozzi medium, gram-positive rods with subterminally located spores were detected. We confirmed the presence of bacteria in the all samples by multiplex PCR.

## Discussion

Blackleg is considered an infection that occurs when the animals are on pasture due to the abundance of dry grass, with summer-autumn seasonality. Rarely, the disease occurs in winter and spring and is caused by non-compliance with sanitary standards and the failure to administer preventive vaccinations (7, 17, 29).

According to classical model, spores of *C. chauvoei* present in contaminated pastures are ingested and undergo one or more replication cycles in the intestine before being absorbed through the intestinal mucosa to the bloodstream and/or being excreted in feces (34–37). When anaerobic conditions are created in areas where the spores are present, most frequently related to trauma (include, injection, etc.), those latent spores germinate, proliferate, and release toxins that produce the clinical manifestations and lesions of blackleg (8, 9, 38, 39). The predisposing factors for lesions of blackleg have not been definitively determined, but it has been suggested that trauma, or stress associated with yarding and management procedures that might result in increased levels of plasma cortisol and catecholamines, may lead to this form of the disease (40, 41).

This outbreak occurred in the spring, when there was snow cover in Siberia. It occurred on cluttered farmland when there were susceptible cattle in the herd, including pregnant cows. This outbreak occurred due to the presence of inappropriate conditions resulting in trauma.

We suppose that trauma that occurred during the handling necessary for the vaccinations by the veterinarian (46.5% of the animals), mechanical papilloma injuries (41.8% of animals) and trauma that occurred during birth (11.6% of animals) resulted in the activated of the pathogen into the cattle and the subsequent development of blackleg. We excluding the vaccine as a source of live *C. chauvoei* because a formol-inactivated vaccine was used for vaccination. Consequently, the factors of infection were cutaneous wounds and tears in the mucous membranes of the reproductive tract. This complements the common understanding of the factors of infection in cases of blackleg (5, 8, 9).

The clinical picture in the examined animals agreed with previously published data. The characteristic features were depression-like behavior, refusal to eat, and gross swelling in the extremities, chest, abdomen and back (21, 22). Abortions and stillbirths were reported in fecund cows. Distinctive features of the described cases were the body temperature, which increased up to 38–39.5°C, and lack of crepitus under pressure in the area of oedema. Previously in published literature, no information could be found about the ocular bleeding in infected animals. Papillomas were the factor of infection both while calving (papillomas vulva) and while feeding (papillomas on the muzzle). Tissue damage from papillomas resulted in an open route of infection and the development of the vegetative forms of *C. chauvoei*. Papillomatoses may lead to infection with *C. chauvoei* spores.

Some animals showed the formation of extensive haematomas containing blood with erythrocytes with basophilic granularity. No such cases have been described before. We believe the formation of haematomas was due to the increased vascular permeability. The appearance of basophilic granularity was due to the with systemic infection of the body and intoxic (34).

When the pathological and anatomical investigations were performed on the carcasses of the cows, the following changes were noted: serous haemorrhagic oedema and emphysema of the subcutaneous tissue and skeletal muscles, serous haemorrhagic lymphadenitis of the regional lymph nodes, and hyperaemia of the organs in the chest and head. The presence of hemorrhages on the serous and mucous membranes of the dead animals, as well as hemorrhages on the internal organs of the fetuses, indicates both systemic infection of the animal and intrauterine fetal infection (10).

Cardiac lesions are mainly characterized by necrohemorrhagic myocarditis, which is consistent with blackleg and distinguishes it from malignant edema. Vascular degeneration and necrosis, with many blood vessels and containing fibrin thrombi, is a prominent feature of blackleg. Vascular changes are more prevalent in cardiac lesions. The histologic lesions in of heart of blackleg are agreed with previously published data. However, adrenal glands lesions have not been previously reported. In our study, we showed thrombi in the vessels and vacuolization in the tissue of adrenal glands, which complements the data on the microscopic pathology of the blackleg.

There are no available reports of some of the gross pathological, such as pulmonary atelectasis, abdominal anemia. But there is evidence of such lung injuries as interstitial pneumonia, edema, leukocytic infiltrations and emphysema were the main lesions in lung tissues of infected animals (25). These signs may be considered unusual for this infection, and further investigation of the manifestation of the infection under different conditions is needed.

The diagnosis of blackleg was confirmed by laboratory studies. The Kitt-Tarozzi medium yielded characteristic growth with gas formation. Gram-positive C rods were found in smears of cultures obtained from infected and dead animals.

Vaccination against *C. chauvoei* is a routine practice in most cattle operations around the world, and close to 100% efficacious in preventing blackleg after natural exposure. However, the efficacy of vaccination to prevent blackleg after experimental challenge was reported to be 50–100%, depending on the dose of the inoculum (31, 42). This could be the explanation for the of cases that occurred in confirmed vaccinated animals in our study.

## Conclusions

An outbreak of blackleg occurred in Siberia in the early spring when the soil was covered with snow, which is not typical for the seasonality of the blackleg. Some unvaccinated cattle and some cattle that had been vaccinated in the previous 2 days developed blackleg. The distinctive clinical signs of the described cases were a subfebrile body temperature and the absence of crepitus when the oedema was depressed. In some animals, the formation of extensive haematomas containing blood with erythrocytes with basophilic granularity was noted. Macroscopic and microscopic lesions are characteristic of the blackleg and are due to *C. chauvoei* histotoxicity and systemic intoxication of the body. Evidence suggests that blackleg can present with a variety of conditions and characteristics in cattle. More research is needed on the various manifestations of blackleg.

## Data Availability Statement

The original contributions presented in the study are included in the article/supplementary material, further inquiries can be directed to the corresponding author/s.

## Author Contributions

AB: conceptualization, data curation, and supervision. AB, II, and OZ: methodology. OB and AB: validation. AB and OZ: formal analysis. OZ and NT: investigation. OB, OZ, and AB: writing—original draft preparation and writing—review and editing. OZ: visualization. AB and II: project administration. All authors have read and agree to the published version of the manuscript.

## Funding

This work was supported by the Federal Research Center for Virology and Microbiology for government assignments N°FGNM-2021-0004.

## Conflict of Interest

The authors declare that the research was conducted in the absence of any commercial or financial relationships that could be construed as a potential conflict of interest.

## Publisher's Note

All claims expressed in this article are solely those of the authors and do not necessarily represent those of their affiliated organizations, or those of the publisher, the editors and the reviewers. Any product that may be evaluated in this article, or claim that may be made by its manufacturer, is not guaranteed or endorsed by the publisher.
